# Insights into the Impact of Linker Flexibility and Fragment Ionization on the Design of CK2 Allosteric Inhibitors: Comparative Molecular Dynamics Simulation Studies

**DOI:** 10.3390/ijms19010111

**Published:** 2018-01-01

**Authors:** Yue Zhou, Na Zhang, Xiaoqian Qi, Shan Tang, Guohui Sun, Lijiao Zhao, Rugang Zhong, Yongzhen Peng

**Affiliations:** 1 National Engineering Laboratory for Advanced Municipal Wastewater Treatment and Reuse Technology, Engineering Research Center of Beijing, Beijing University of Technology, Beijing 100124, China; zhouyue2016@bjut.edu.cn; 2 Beijing Key Laboratory of Environmental & Viral Oncology, College of Life Science and Bioengineering, Beijing University of Technology, Beijing 100124, China; 1992346688@emails.bjut.edu.cn (X.Q.); tangshan@emails.bjut.edu.cn (S.T.); sunguohui@bjut.edu.cn (G.S.); zhaolijiao@bjut.edu.cn (L.Z.); lifesci@bjut.edu.cn (R.Z.)

**Keywords:** protein kinase CK2, allosteric inhibitor, fragment-based design, αD region, linker

## Abstract

Protein kinase is a novel therapeutic target for human diseases. The off-target and side effects of ATP-competitive inhibitors preclude them from the clinically relevant drugs. The compounds targeting the druggable allosteric sites outside the highly conversed ATP binding pocket have been identified as promising alternatives to overcome current barriers of ATP-competitive inhibitors. By simultaneously interacting with the αD region (new allosteric site) and sub-ATP binding pocket, the attractive compound CAM4066 was named as allosteric inhibitor of CK2α. It has been demonstrated that the rigid linker and non-ionizable substituted fragment resulted in significant decreased inhibitory activities of compounds. The molecular dynamics simulations and energy analysis revealed that the appropriate coupling between the linker and pharmacophore fragments were essential for binding of CAM4066 with CK2α. The lower flexible linker of compound 21 lost the capability of coupling fragments A and B to αD region and positive area, respectively, whereas the methyl benzoate of fragment B induced the re-orientated Pre-CAM4066 with the inappropriate polar interactions. Most importantly, the match between the optimized linker and pharmacophore fragments is the challenging work of fragment-linking based drug design. These results provide rational clues to further structural modification and development of highly potent allosteric inhibitors of CK2.

## 1. Introduction

Protein kinases play predominant regulatory roles in nearly every aspect of cellular function, and have been considered as one of the most attractive drug targets [1,2]. Some FDA-approved anti-cancer drugs of protein kinase, such as Gefitinib, Palbociclib, and Tofacitinib, are ATP-competitive inhibitors. However, most of these inhibitors targeting highly conserved ATP-binding pocket of the protein kinase family were impeded from the clinical-used drugs due to the risk of the off-target and side effects [3,4,5]. It is urgent to discover allosteric inhibitors targeting novel druggable sites outside the catalytic box. Given the over-expression in a range of cancer cell lines, protein kinase CK2 has been regarded as a representative of cancer therapeutic targets [6,7,8]. To date, many ATP-competitive inhibitors with different scaffolds have been developed, such as benzimidazole derivatives, anthraquinone derivatives, tricyclic quinolone derivatives and natural products [9,10,11]. Despite their anti-proliferation effects on malignant tumors, these inhibitors are still facing a stumbling block of lower specificity and diversity deficiencies to be rejected as clinical drugs [12,13,14]. Therefore, it is a challenging task to develop inhibitors with higher affinity and selectivity by targeting the allosteric sites, which may avoid the drawbacks of most conventional ATP-competitive kinase inhibitors. For instance, DRB and W16 have been identified as allosteric inhibitors of CK2. Both are able to disrupt the tetrameric assembly at the CK2α/β interface [15,16]. Recently, Jiang et al. identified another four potential allosteric pockets of CK2α and predicated allosteric pathways between allosteric sites and the active site through bioinformatics methods, such as site 2 (Leu249, Trp281 and tye307), site 3 (Tye125, Met225 and Leu128) and site 4 (His148, Tyr211 and Thr314) as well as site 5 (Trp33, Lys75, Ile78 and Pro109) [17]. These potent sites inspire researchers to design and synthesize the novel allosteric inhibitors of CK2α.

Among these allosteric sites, site 3 is located in the αD region next to the ATP binding pocket. The αD region of CK2α presents the unique features in contrast to other protein kinases, including much more flexibility, partially open conformation different from the closed status of other kinases and the non-conserved residues throughout the whole CMGC family, which includes cyclin-dependent kinases (CDKs), mitogen-activated protein kinases (MAPKs), glycogen synthase kinases (GSKs), and CDK-like kinases (CLKs), and thus appears to be an attractive site for the novel selective CK2 allosteric inhibitors design [17,18,19]. Recently, Brear and Fusco et al. discovered a new potent inhibitor CAM4066 that targeted a previously unseen pocket “αD pocket” and ATP site, simultaneously using the fragments linking strategy [20]. Notably, the comparison of the structure and inhibitory activity of compounds 21 (IC_50_ = n/a) and Pre-CAM4066 (IC_50_ = n/a) versus compound CAM4066 (IC_50_ = 370 nM) indicated that the inappropriate linker and fragments resulted in a significant reduction of inhibitory activities. Therefore, there is an urgent need to systematically investigate the structural basis for the decreased inhibitory activities of CAM4066 derivatives.

In this study, comparative molecular dynamics (MD) analyses were conducted to examine the detailed binding modes of the three compounds (CAM4066, Pre-CAM4066, and compound 21) to CK2α. In addition, molecular mechanics Poisson–Boltzmann surface area (MM/PBSA) binding free-energy calculations were performed to elucidate the instability of the compound 21 and Pre-CAM4066 systems. Our findings may provide valuable information for further structural modification and development of highly potent allosteric inhibitors of CK2.

## 2. Results

### 2.1. Molecular Dynamics Simulation Studies

#### 2.1.1. Overall Features of Dynamic Behaviors

**Figure 1:**
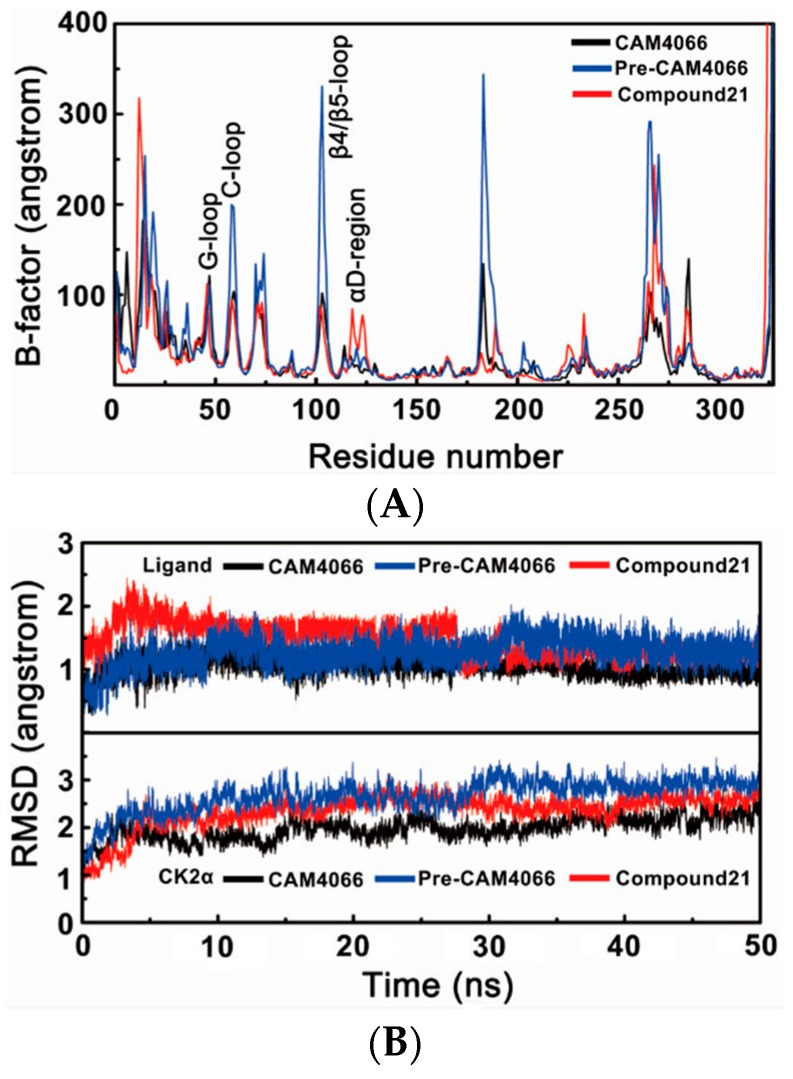
(**A**) The time dependence of RMSD of inhibitors (**upper**) and CK2α (**lower**) for CK2 in complex with CAM4066, Pre-CAM4066 and compound 21; and (**B**) calculated per-residue B-factor of three complexes systems.

The dynamic stability and behavior of three systems were explored by 50 ns conventional MD simulations in explicit water. Figure 1A shows the time-dependent root mean square deviation (RMSD) profile of each trajectory from the minimized structure. The relatively stable RMSD values during the last 20 ns indicated the approaching acceptable equilibrium state. As depicted in the lower panel, the fluctuations of RMSD data of CK2α in all systems followed the similar trend throughout the simulations. The average RMSD values of compound 21 (2.5 Å) and pre-CAM4066 (3.0 Å) systems were higher than those of CK2α–CAM4066 complex (2.0 Å), which suggested that the CK2α structures in the two systems underwent conformational changes to a certain extent. In contrast, some slightly different behaviors could be observed in the upper panel. The RMSD values of CAM4066 remained around 1.0 Å during the MD simulation, while the corresponding data of compound 21 and Pre-CAM4066 gradually reduced from 2.0 Å to 1.5 Å and increased from 1.0 Å to 1.5 Å, respectively. It was speculated that rigid linker of compound 21 and non-ionizable fragment B of Pre-CAM4066 resulted in the inappropriate interactions between ligands and CK2, which induced that the two compounds may deviate away from its original position.

To identify the detailed flexible regions in the protein structure during MD simulation, the thermodynamic stability of the three systems characterized by B-factor are shown in Figure 1B. Judging from the high B-factor values in the region of the N-terminal, G-loop, β4/β5-loop and C-terminal for three systems, these residues appeared to exhibit large fluctuations, as observed in other CK2α-ligand complexes, which have been demonstrated in previous studies [19,21,22,23]. It was speculated that the higher B-factors of αD/hinge region of compound 21 system resulted from the varied binding of fragment A. For Pre-CAM4066 system, as a consequence of the decreased interactions between methyl benzoate substituent and Lys68, the C-loop next to the positive area was more flexible in contrast to other systems.

#### 2.1.2. Comparative Analysis of Different Binding Modes of Three Compounds

**Figure 2:**
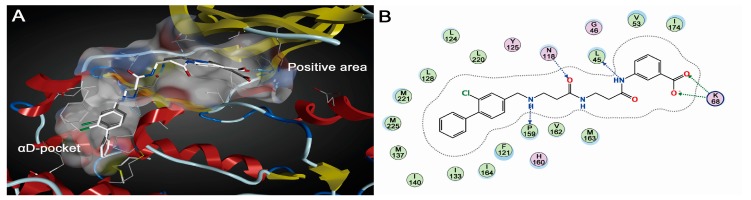
(**A**) Superimposition of co-crystallized pose (gray stick) and the average structure (white stick) of CAM4066; and (**B**) interactions between CAM4066 and the residues in the active site.

As indicated in the crystallized conformation, with the flexible *N*-(3-oxobutyl)butyramide scaffold as a bridge, CAM4066 simultaneously extended to the positive area of ATP binding pocket and the allosteric site αD region by forming hydrophobic and polar interactions. MD simulation provided the detailed dynamical behaviors of the CK2α-CAM4066 complex (Figure 2). Fragment A was entrapped into the cavity of αD region, where the biphenyl group formed stable hydrophobic packing with side chains of residues Phe121, Leu124, Tyr 125, Leu128, Ile133, Tyr136, Met137, Ile140, Met221 and Met225. The alternative H-bonds between Pro159 (66.59% occurrence) and Val162 (63.87% occurrence) and the NH of fragment A were considered to be an anchor for the binding of fragment A into αD pocket. The carboxylate substituent of fragment B tended to create electrostatic interactions with residue Lys68 of positive area. In contrast to the crystallized CK2α-CAM4066 complex, besides the subtle positional deviation of the linker, the new H-bond (62.54% occurrence) between the NH group of fragment B and the carbonyl oxygen atom of Leu45 was identified. It is speculated that the stable polar interactions between fragment B and Lys68 induced the reorientation of O3 and O4 atoms of the linker, which resulted in the pairing H-bonds between N2 and O3 of linker and Asn118 replaced by the H-bond between O4 atom of linker and NH2 of Asn118 (32.73% occurrence).

**Figure 3:**
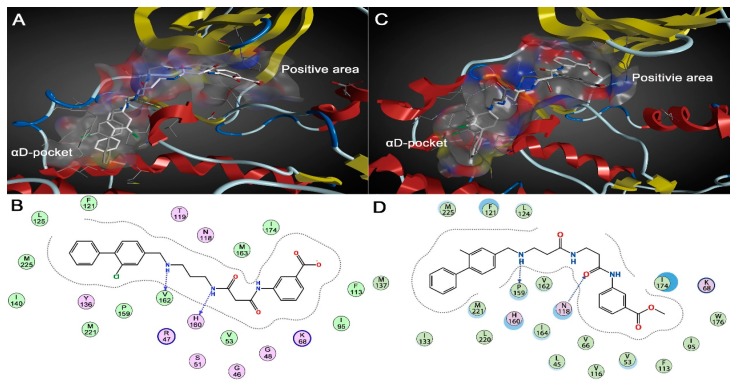
(**A**) Superimposition of co-crystallized pose (gray stick) and the average structure (white stick) ofcompound 21; (**B**) stable binding mode of compound 21 with the residues in the active site; (**C**) superimposition of co-crystallized pose (gray stick) and the average structure (white stick) of compound Pre-CAM4066; and (**D**) stable binding mode of compound Pre-CAM4066 with the residues in the active site.

**Figure 4:**
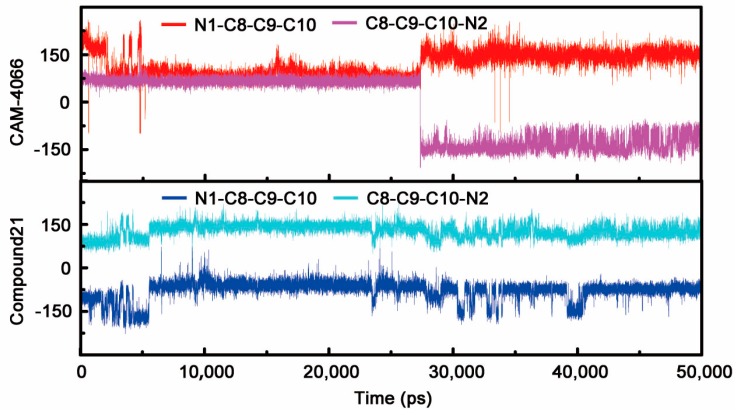
The dihedral N1-C8-C9-C10 and C8-C9-C10-N2 as the time evolution of CAM4066 and compound 21.

Given the limited shortcomings of X-ray crystallization method, the static conformation of compound 21 could not unveil the underlying interaction mechanism with CK2α. Owing to the development of molecular modeling methods, MD analysis can be used to interpret and complement the experimental phenomena. As shown in Figure 3A,B, although compound 21 occupied the same binding pocket as CAM4066 did, the twisted linker and re-orientated fragments A and B can be found easily. In the complex of CK2α-CAM4066, the flexible linker had the capability to couple fragment A and B binding into the αD region and positive area, respectively. With the replacement of ethyl with the methyl group, the rigid linker of compound 21 was incapable of capturing enough conformation to guarantee two fragments targeting two regions simultaneously as the flexible linker of CAM4066 could. It was also supported by the dihedrals angles analysis shown in the Figure 4. During the last 20 ns, the dihedral angles of N1-C8-C9-C10 and C8-C9-C10-N2 of compound 21 were kept fixed near −75° and 150°, respectively, whereas the corresponding two dihedral angles of CAM4066 presented cooperative rotation behaviors. It could be concluded that the flexible linker of CAM4066 covered more chemical spaces than the compound 21 did. The N2 atom of the twisted linker established a stable H-bond with the carbonyl oxygen atom of His160 (85.84% occurrence). Meanwhile, fragment B was also deprived of the ability to make polar interactions with positive area. Fragment A was still entrapped into the hydrophobic pocket of αD region by the alternative H-bond among N3 atom, Val162 (78.70% occurrence) and Pro159 (41.08% occurrence).

As only the methyl benzoate of fragment B is the subtle difference between Pre-CAM4066 and CA4066, the position and orientation of Pre-CAM4066 was similar to those of CAM4066 except the deviated fragment B. As shown in Figure 3C,D, the linkers of two compounds were nearly overlapped. Fragment A of Pre-CAM4066 still located in αD pocket by forming the stable H-bonds with Pro159 and Val162 (71.98% and 60.35% occurrence, respectively), whereas the methyl benzoate of fragment B could not make any interactions with Lys68 and rotated out of the positive area. As triggered by the movement of fragment B, a stable H-bond was established between O3 atom of the deviated linker and NH2 group of Asn118 (85.70% occurrence). To sum up, both the flexible linker and ionizable substituent of fragment B are two determinants for binding of CAM4066. As indicated from the stable interactions between fragment A and αD region of three systems, a fact worth highlighting is that fragment A should be identified as a new pharmacophore group of αD region.

### 2.2. Energy Analysis

To elucidate the quantitative effect of flexible linker and ionizable substituted fragment B on the compounds binding affinity, molecular mechanics/Poisson−Boltzmann surface area (MM/PBSA) energy terms for each system were calculated, as listed in Table 1. The total ∆*G*_binding_ of the CAM4066 (IC_50_ = 0.370 μM, *K_d_* = 0.320) was −54.68 kcal/mol, which was 3.25 and 6.51 kcal/mol lower than those of compound 21 (IC_50_ = n/a, *K_d_* = 1.64) and Pre-CAM4066 (IC_50_ = n/a, *K_d_* = n/a), respectively. These data indicated that CAM4066 exhibited the highest affinity binding to CK2. Further analysis of the energy components responsible for the binding free energies showed that ∆*E*_ele_, ∆*E*_vdw_, and ∆*E*_non-polar_ was the main driving forces for the three systems. The ∆*E*_ele_ values of compound CAM4066 (−110.41 kcal/mol) exhibited more favorable contributions than those of compound 21 (−53.42 kcal/mol) and Pre-CAM4066 (−11.17 kcal/mol), which was in accordance with the loss of electrostatic interactions between fragment B and the positive area of two systems. Taking the polar contribution of ∆*G*_polar_ into consideration, the total electrostatic interaction energy (∆*G*_ele_) was positive for three compounds. This finding may be interpreted by the fact that the favorable contribution of the ∆*E*_ele_ was more than compensated by the ∆*G*_polar_ upon binding. Thus, ∆*G*_polar_ was unfavorable to this class of complexes.

## 3. Discussion

Fragment-based drug discovery (FBDD) is a robust tool for identify new anti-cancer leads and clinical-used drugs, such as Vemurafenib and Venetoclax [24]. Rather than screening millions of compounds as conducted by high-throughput screening, FBDD begins with the collection of smaller compounds or fragments with high affinity to favorable spots [25]. The small size of the fragment library is much more efficient in covering the chemical spaces than the amount of compound library does [26]. Hence the fragment collection is the most fundamental step for FBDD.

Fusco et al. [20] obtained two effective fragment libraries for the unreported αD site and positive area of ATP sub-pocket with the experimental screening method. As the αD site cavity consisted of hydrophobic residues Phe121, Leu124, Tyr125, Leu128, Ile133, Tyr136, Met137, Ile140, Met221 and Met225, and was also highly solvent accessible, hydrophobic fragments were entrapped into the cavity of αD region and formed two pair H-bonds with carbonyl oxygen atom of Pro159 and Val162, respectively. Meanwhile, these fragments also make the contribution to the selectivity of compounds by specific interacting with αD site rather than the hinge region of ATP binding pocket. Recently, there is increasing interests in the identification of pharmacophore fragments using efficient and economical virtual splitting and screening technologies. In our study, the reported co-crystallized ATP-competitive inhibitors were decomposed into three types of fragments targeting CK2α ATP binding sub-pocket (hydrophobic, hinge and positive area) by a pharmacophore oriented fragmentation algorithm (unpublished). The pharmacophore fragment library targeting sub-binding pocket would enrich the ATP site fragment database and provide structural elements for the rational design of CK2 inhibitor. Similarly, Zhang and Zhao discovered of a novel series of CK2 inhibitors by using a library of virtual fragments with key functionalities via fragmentation of bioactive molecules [27].

Fragment linking is the useful strategy of FBDD, in which two fragments that bind to a distinct site are joined together by the suitable linker. Thus, the length and conformational flexibility of the linker are two decisive factors that influence the binding of fragments. A serial of linkers were obtained by optimization of the alkyl chain and polar groups, and the (*N*-(3-oxobutyl)butyramide) was chosen as the best candidate. Besides the H-bonds between the best linker and residue Asn118, this linker also had no negative effects on the conformation and binding modes of fragment A and B. This suggested that the linker of CAM4066 met the requirement proposed by Kim [25]. Any variation exiting in the flexible linker may exert the detrimental effect on the binding modes of compounds. For instance, the rigid linker with methyl group of compound 21 destroyed the original proper interactions the αD region and positive area of CK2α t as CAM4066 did. Furthermore, chemical environment and structural topology of two sub-pockets were two other key factors for linker optimization.

## 4. Materials and Methods

### 4.1. System Setup and Parameters Preparation

The atomic co-ordinates of CK2α-CAM4066 and CK2α-compound 21 were retrieved directly from the Protein Data Bank [PDB ID: 5CU4 and 5MO8] and hetero atoms, expected for water molecules within 6.5 Å of the ligand, were removed [18,20]. The compound Pre-CAM4066 was constructed based on the compound CAM4066 by using the SYBYL 8.1 program and the Tripos force field was used to energetically minimize the compound [28,29]. The partial atomic charges of three compounds were obtained via quantum electronic structure calculations including an optimization procedure using the Gaussian 03 program at the HF/6-31G* level, electrostatic potential (ESP) generation using the MerzSingh–Kollman van der Waals parameters, the atom-centered charge fitting through the RESP program implemented in the AMBER 10 package [30,31,32]. Subsequently, each system was neutralized by adding suitable counterions and then solvated in a truncated octahedral box of TIP3P water molecules [33,34]. The chemical structures and IC_50_ values of three compounds are listed in the Table 2.

### 4.2. Molecular Dynamics Simulations

Molecular dynamics simulations were initiated on the CAM4066, Pre-CAM4066 and compound 21 and each simulation was performed for 50 ns using the Amber 10 package [35]. The force field parameters for protein and ligands were calculated by the AMBER FF03 force field and the general AMBER force field (GAFF), respectively [36,37]. First, the geometric strain and close intermolecular contacts were relieved in the energy minimizations using the steepest descent and conjugate gradient methods. Second, each energy-minimized structure was gradually warmed from 0 to 300 K with weak constraint to the complex (5.0 kcal/mol) over 15 ps, followed by constant temperature equilibration at 300 K for 35 ps with constant volume dynamics. Third, MD simulations were carried out with the periodic boundary condition in the NPT ensemble, using a non-bonded cutoff of 10 Å to truncate the VDW non-bonded interactions [38]. Temperature (300 K) and constant pressure (1 atm) were maintained by Langevin dynamics temperature coupling with a time constant of 1.0 ps and isotropic position scaling with a relaxation time of 2.0 ps, respectively. The long-range electrostatic interactions were calculated based on the particle-mesh Ewald (PME) algorithm, and the SHAKE algorithm was applied to constrain all bonds involving hydrogen atom [39,40].

### 4.3. MM/PBSA Calculations

The MM-PBSA methods was employed to evaluate the three compounds binding energies and the effects of flexibility of linker and ionizable substituted fragment on the compounds binding from an energetic view [41,42]. For each system, the binding energy (Δ*G*_binding_) was calculated for the configurations taken from a single trajectory based on the following equation:

Δ*G*_binding_ = *G*_complex_ − (*G*_protein_ + *G*_ligand_) = Δ*E*_gas_ + Δ*G*_sol_ − *T*Δ*S*

where the gas molecular mechanical energy (Δ*E*_gas_) is calculated as a sum of internal energies (i.e., bond, angle, and dihedral), van der Waals (*E*_vdw_) and electrostatic energies (*E*_ele_) using the SANDER module without applying a cutoff for non-bonded interactions. The solvation free energy (Δ*G*_sol_) is composed of electrostatic (Δ*G*_polar_) and non-polar (Δ*G*_non-polar_) contributions. The electrostatic contribution to the solvation free energy (Δ*G*_polar_) is determined by PB model as implemented in SANDER, applying dielectric constants of 1 and 80 to represent the solute and the exterior medium phases, respectively. The non-polar component (Δ*G*_non-polar_) is calculated using a linear function of solvent-accessible surface area (SASA) as follows: Δ*G*_non-polar_ = *λ*·SASA + *b*, where the corresponding parameters λ and b are set to 0.00542 kcal/(mol Å^2^)and 0.92 kcal/mol, respectively [43]. Given the large computational overhead and low prediction accuracy, the time consuming conformational entropy change (−*T*Δ*S*) was not considered [44,45]. The entropy term has been neglected, assuming that it will be very similar for all of the systems.

## 5. Conclusions

MD simulations and energy calculations were performed to elucidate the structural mechanisms through which the rigid linker and non-ionizable substituted fragment influence binding affinity. It seemed that the optimized linker was not only the bridge of the two pharmacophore fragments, but also the adjustor for the binding of fragments into sub-pockets. Both the linker of compound 21 and fragment B of Pre-CAM4066 could not form the proper interactions with CK2α as those of CAM4066, whereas fragment A of three systems maintained stable interactions with αD region of CK2α. In addition, the energy analysis enabled the qualitative investigation of the effect of flexible linker and ionizable substituted fragment B on the three complexes. This will provide the theoretical basis and experiment guidance for the development of potent allosteric inhibitors of CK2.

## Figures and Tables

**Table 1:** Energy terms of MM/PBSA results for three CK2α-inhibitor complexes systems.

Energy Term (kcal/mol)	CAM4066	Compound 21	Pre-CAM4066
Δ*E*_ele_	−110.41 ± 2.83	−53.42 ± 3.87	−11.17 ± 4.26
Δ*E*_vdw_	−75.00 ± 3.16	−65.18 ± 3.64	−68.35 ± 4.09
Δ*E*_gas_^a^	−185.41 ± 5.23	−118.60 ± 3.64	−79.52 ± 4.52
Δ*G*_nonpolar_	−8.56 ± 0.13	−8.29 ± 0.19	−8.89 ± 0.18
Δ*G*_polar_	139.29 ± 4.18	75.46 ± 4.42	40.24 ± 2.94
Δ*G*_sol_^b^	130.72 ± 3.15	67.16 ± 3.36	31.35 ± 2.96
Δ*G*_ele_^c^	28.88 ± 5.32	22.04 ± 3.15	29.07 ± 3.98
Δ*G*_binding_^d^	−54.68 ± 4.87	−51.43 ± 4.42	−48.17 ± 3.33
ΔΔ*G*_binding_	0	3.25	6.51

^a^ Δ*E*_gas_ = Δ*E*_ele_ + Δ*E*_vdw_; ^b^ Δ*G*_sol_ = Δ*G*_polar_ + Δ*G*_nonpolar_; ^c^ Δ*G*_ele_ = Δ*E*_ele_ + Δ*G*_polar_; ^d^ Δ*G*_binding_ = Δ*E*_ele_ + Δ*E*_vdw_ + Δ*G*_sol_.

**Table 2:** Chemical structures, *K_d_* and IC_50_ values of CAM4066, Pre-CAM4066 and compound 21.

Compound	Fragment A	Linker	Fragment B	IC_50_ (μM)	*K_d_*	PDB
CAM4066	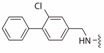	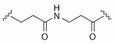	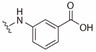	0.370	0.320	5CU4
Pre-CAM4066	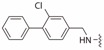	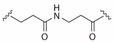	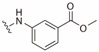	n/a	n/a	n/d
21	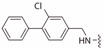	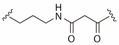	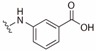	n/a	1.64	5MO8

n/a = not active; n/d = not determined.
